# Influence of partial and complete glutamine-and glucose deprivation of breast-and cervical tumorigenic cell lines

**DOI:** 10.1186/s13578-015-0030-1

**Published:** 2015-07-08

**Authors:** Michelle Helen Visagie, Thandi Vuyelwa Mqoco, Leon Liebenberg, Edward Henry Mathews, George Edward Mathews, Anna Margaretha Joubert

**Affiliations:** Department of Physiology, University of Pretoria, Private Bag X323, Arcadia, 0007 South Africa; Centre for Research and Continued Engineering Development, North-West University, Lynnwood Ridge, South Africa

**Keywords:** Metabolism, Apoptosis, Morphology, Reactive oxygen species, Glycolysis, Glutamine, Glucose

## Abstract

**Background:**

Due to their high proliferative requirements, tumorigenic cells possess altered metabolic systems whereby cells utilize higher quantities of glutamine and glucose. These altered metabolic requirements make it of interest to investigate the effects of physiological non-tumorigenic concentrations of glucose and glutamine on tumorigenic cells since deprivation of either results in a canonical amino acid response in mammalian cell.

**Methods:**

The influence of short-term exposure of tumorigenic cells to correlating decreasing glutamine- and glucose quantities were demonstrated in a highly glycolytic metastatic breast cell line and a cervical carcinoma cell line. Thereafter, cells were propagated in medium containing typical physiological concentrations of 1 mM glutamine and 6 mM glucose for 7 days. The effects on morphology were investigated by means of polarization-optical transmitted light differential interference contrast. Flow cytometry was used to demonstrate the effects of glutamine-and glucose starvation on cell cycle progression and apoptosis induction. Fluorometrics were also conducted to investigate the effects on intrinsic apoptosis induction (mitocapture), reactive oxygen species production (2,7-dichlorofluorescein diacetate) and acidic vesicle formation (acridine orange).

**Results:**

Morphological data suggests that glutamine-and glucose deprivation resulted in reduced cell density and rounded cells. Glutamine-and glucose starvation also resulted in an increase in the G_2_M phase and a sub-G_1_ peak. Complete starvation of glutamine and glucose resulted in the reduction of the mitochondrial membrane potential in both cell lines with MDA-MB-231 cells more prominently affected when compared to HeLa cells. Further, starved cells could not be rescued sufficiently by propagating since cells possessed an increase in reactive oxygen species, acidic compartments and vacuole formation.

**Conclusion:**

Starvation from glutamine and glucose for short periods resulted in decreased cell density, rounded cells and apoptosis induction by means of reactive oxygen species generation and mitochondrial dysfunction. In addition, the metastatic cell line reacted more prominently to glutamine-and glucose starvation due to their highly glycolytic nature. Satisfactory cellular rescue was not possible as cells demonstrated oxidative stress and depolarized mitochondrial membrane potential. This study contributes to the knowledge regarding the *in vitro* effects and signal transduction of glucose and/or l-glutamine deprivation in tumorigenic cell lines.

## Introduction

Tumorigenic tissue possesses altered metabolic activities when compared to differentiated, non-proliferating tissue. These modified metabolic activities exerted by the tumors are necessary for the highly proliferative nature of transformed-and tumorigenic tissue [1]. In tumorigenic cells, a shift occurs from adenosine triphosphate (ATP) production through phosphorylation to generation by means of glycolysis even in the presence of oxygen [2]. Cancer-associated aerobic glycolysis which results in the production of lactic acid and pyruvate was described nearly 100 years ago and is known as the Warburg effect [2, 3]. Initially it was reported that the effect was due to mitochondrial dysfunction, however, further research indicates that cancer cells prefer glucose catabolism via glycolysis over oxidative phosphorylation even if the mitochondria is competent [3]. Since glucose is thus mainly utilized for glycolysis, glutamine is used as the mitochondrial tricarboxylic acid cycle (TCA) substrate and for nicotinamide adenine dinucleotide phosphate (NADPH) and fatty acids synthesis [4].

Besides glycolysis, tumorigenic cells are also addicted to glutaminolysis for proliferation purposes. Glutaminolysis is defined as the conversion of glutamine to glutamate. This process provides carbon and nitrogen used for the production of energetic, biosynthetic and reductive precursor for tumorigenic cells [5]. Thus, tumorigenic cells clearly utilize higher quantities of glutamine and glucose when compared to non-tumorigenic and senescent cells.

Glucose and glutamine are the most crucial building blocks required for metabolism and subsequent proliferation and tumorigenic processes [1]. However, due to the largely different metabolic requirements of tumorigenic cells it is of interest and importance to investigate the physiological effects of non-tumorigenic concentrations of glucose and glutamine on tumorigenic cells. This is especially significant since deprivation of either results in a canonical amino acid response (ASS) in mammalian cells [6]. In addition, gene expression studies conducted on primary and metastatic tumors demonstrated that metastatic cells are more prominently dependent on glycolysis when compared to oxidative phosphorylation [7].

This study thus investigated the influence of correlating decreasing quantities of glutamine and glucose on morphology, mitochondrial membrane potential, reactive oxygen species (ROS) and acidic vesicle formation after short-term exposures (2 h, 4 h, 6 h) in a highly glycolytic estrogen receptor metastatic negative breast cell line and a cervical carcinoma cell line. In addition, after exposure, medium was replaced with medium containing a typical physiological concentration of glutamine-and glucose (1 mM and 6 mM respectively) whereby cells were allowed to proliferate for 7 days after which the influence of this exposure was also investigated.

## Materials and methods

### Cell lines

Human cervical adenocarcinoma cell line (HeLa) and a highly metastatic breast adenocarcinoma cell line (MDA-MB-231) were chosen to demonstrate the effects of medium consisting of varying metabolic states. The HeLa cell line was purchased through Sterilab Services (Pty) Ltd, Johannesburg, South Africa, from the American Tissue Culture Collection (ATCC), Maryland, United States of America. The HeLa cell line is the oldest and most distributed immortalized cell line that presents with aggressive growth and doubles on average every 24 h [8]. The primary source of energy in HeLa cells is glutamine rather than glucose, thus demonstrating that oxidative phosphorylation is preferential to generate ATP [9]. Furthermore, HeLa cells are capable of adapting their mitochondrial network, structure and function depending on substrate basis to generate energy exclusively from oxidative phosphorylation by remodelling its mitochondria [10].

The MDA-MB-231 cell line was provided by Microsep (Pty) Ltd Johannesburg, South Africa. MDA-MB-231 is a triple negative tumorigenic breast cell line. This indicates that MDA-MB-231 cells do not express receptors for steroid hormones (estrogen and progesterone), type II receptor tyrosine kinase (RTK) Her-2, but do possess upregulation of basal cytokeratins and epidermal growth factor response [11–13]. Further, the MDA-MB-231 cell line is highly metastatic and presents with elevated glycolytic activity under normoxic conditions [14]. MDA-MB-231 cells mainly use glycolysis rather than mitochondrial respiration to produce energy required for cell functioning and proliferation [15]. Furthermore, MDA-MB-231 cells contain mitochondria presenting with deoxyribonucleic acid (DNA) mutations resulting in decreased oxidative metabolism [16].

### General reagents

Actinomycin D, glucose, l-glutamine and sodium pyruvate free-Dulbecco’s minimum essential medium eagle (DMEM), as well as high glucose (25.52 mM, 4500 mg/l), l-glutamine (4 mM) and sodium pyruvate (1 mM, 110 mg/l) containing DMEM, bicarbonate, l-glutamine, glucose, trypsin, crystal violet, NaCl, KCl, KH_2_PO_4_ and Na_2_HPO_4_, acridine orange and 2,7-dichlorofluorescein diacetate (DCF-DA) were supplied by Sigma Chemical Co. (St. Louis, United States of America). Heat-inactivated fetal calf serum (FCS), sterile cell culture flasks and plates were obtained through Sterilab Services (Kempton Park, Johannesburg, South Africa). Penicillin, streptomycin and fungizone were purchased from Highveld Biological Ltd (Pty). (Sandringham, Gauteng, South Africa).

### General cell culture procedures

Cells were grown and maintained in 25 cm^2^ tissue culture flasks in a humidified atmosphere at 37 °C, 5 % CO_2_ in a Forma Scientific water-jacketed incubator (Ohio, United States of America). Cells were cultured in DMEM with 25.52 mM glucose, 4 mM l-glutamine, and 1 mM sodium pyruvate supplemented with 10 % heat-inactivated fetal calf serum (56 °C, 30 min), 100 U/ml penicillin G, 100 μg/ml streptomycin and fungizone (250 μg/l). The media according to metabolic state were prepared 24 h before exposure and autoclaved to ensure sterility.

Cells were exposed to varying metabolic conditions as described below:

Control: DMEM with 25.52 mM glucose, 4 mM l-glutamine, and 1 mM sodium pyruvate supplemented with 10 % heat-inactivated FCS (56 °C, 30 min), 100 U/ml penicillin G, 100 μg/ml streptomycin and fungizone (250 μg/l).

Experimental Condition 1: DMEM with 6 mM glucose, 1 mM l-glutamine, and 0 mM sodium pyruvate supplemented with 10 % heat-inactivated FCS (56 °C, 30 min), 100 U/ml penicillin G, 100 μg/ml streptomycin and fungizone (250 μg/l).

Experimental condition 2: DMEM with 3 mM glucose, 0.5 mM l-glutamine, and 0 mM sodium pyruvate supplemented with 10 % heat-inactivated FCS (56 °C, 30 min), 100 U/ml penicillin G, 100 μg/ml streptomycin and fungizone (250 μg/l).

Experimental Condition 3: DMEM with 0 mM glucose, 0 mM l-glutamine, and 0 mM sodium pyruvate supplemented with 10 % heat-inactivated FCS (56 °C, 30 min), 100 U/ml penicillin G, 100 μg/ml streptomycin and fungizone (250 μg/l).

Positive control: Growth medium containing 0.1 μg/ml actinomycin D was used as a positive control to induce cell death via apoptosis.

General experimental procedures for short term exposures and recovery experiments:

Short term exposure: Cells were seeded at 500 000 cells per 25 cm^2^ flask or 5 000 cells/well into Nunc F96 microwell plates (AEC-Amersham Soc (Ltd), Kyalami, South Africa). After 24 h, cells were exposed to varying metabolic conditions for 2 h, 4 h and 6 h. Thereafter, all experimental methods were conducted as described below.

Recovery experiment: Cells were seeded at 60 000 cells per 25 cm^2^ flask or 850 cells/well into Nunc F96 microwell plates (AEC-Amersham Soc (Ltd), Kyalami, South Africa). After 24 h, cells were exposed to the varying metabolic conditions for 2 h, 4 h and 6 h. Subsequently, cells were washed with PBS and medium was replaced with DMEM containing 6 mM glucose and 1 mM l-glutamine. Medium was subsequently replaced every 2 days. Subsequently, all experimental methods were conducted as described below.

### Polarization-optical transmitted light differential interference contrast

Polarization-optical transmitted light differential interference contrast (PlasDIC) is a contrast method used to view morphology. PlasDIC displays the required phase profile which is relative to the product of the section thickness and the refractive index difference between the environment and the average refractive index of quartz. PlasDIC has high-quality DIC imaging of individual cells, cell clusters and thick individual cells in plastic cell-culture vessels [16, 17]. PlasDIC was conducted according to Visagie, et al. [17].

### Cell cycle progression and apoptosis induction

Flow cytometry was employed to measure the DNA content of cells after exposure to the various metabolic conditions and to monitor the effect on cell cycle progression [18]. The latter was accomplished by means of ethanol fixation and propidium iodide staining which was conducted according to Mqoco, et al. [16]. Cell cycle Propidium iodide fluorescence was measured with fluorescence activated cell sorting (FACS) FC500 System flow cytometer (Beckman Coulter South Africa (Pty) Ltd). Data from at least 10 000–30 000 events were analyzed with CXP software (Beckman Coulter South Africa (Pty) Ltd. (Pretoria, Gauteng, South Africa). Cell cycle distributions was calculated with Cyflogic 1.2.1 released 2008/11/19 (Perttu Terho & Cyflo Ltd) by assigning relative DNA content per cell to sub-G_1_, G_1_, S and G_2_M fractions.

### Mitochondrial membrane potential

A reduction in mitochondrial membrane potential is an early indicator of apoptosis induction [19]. Changes in the mitochondrial membrane potential were investigated using the mitocapture antibody BIOCOM biotech Pty (Ltd) (Clubview, South Africa. Mitocapture is a cationic dye that accumulates in the mitochondria of healthy cells. However, the Mitocapture is unable to accumulate in the mitochondria of apoptotic cells due to the altered mitochondrial membrane potential and therefor the Mitocapture remains in the cytoplasm in its monomer form (green) [20]. After above-mentioned general experimental procedures were followed, diluted Mitocapture solution (mixed according to suppliers’ instructions) was pipetted to all samples. Samples were then incubated for 60 min in a humidified atmosphere (37 °C, 5 % CO_2_). Samples were then incubated the fluorescence was measured at excitation wavelength of 485 nm and emission wavelength of 520 nm using fluorometrics (Department of Pharmacology, University of Pretoria).

### Hydrogen peroxide generation

Hydrogen peroxide generation was measured using 2, 7-dichlorofluorescein diacetate (DCFDA). DCFDA, a non-fluorescent probe, which upon oxidation by ROS and peroxides is converted to the highly fluorescent derivative DCF [21]. After above-mentioned general experimental procedures were followed, DCF-DA (200 μl; 10 μM) was pipetted to all the samples and were subsequently incubated for 60 min in a humidified atmosphere (37 °C, 5 % CO_2_. Fluorescence was measured at excitation wavelength of 485 nm and emission wavelength of 520 nm using fluorometrics (Department of Pharmacology, University of Pretoria).

### Acridine orange staining

Acridine orange is a lysosomotropic fluorescent compound that moves freely across cell membranes when uncharged [22]. However, acridine orange accumulates in its protonated form in acidic compartments and thus serves as a tracer for acidic vesicular organelles including autophagic vacuoles and lysosomes [22]. After above-mentioned general experimental procedures were followed, PBS containing acridine orange (200 μl; 5 mg/ml) was added to all the samples and samples were incubated for 60 min in a humidified atmosphere (37 °C, 5 % CO_2_)_._ Fluorescence was measured at excitation wavelength of 485 nm and emission wavelength of 520 nm using fluorometrics (Department of Pharmacology, University of Pretoria).

### Statistics

At least three independent experiments were conducted for all techniques. Each independent fluorometrical experiment had a sample size of 3. Averages of each experiment were represented in bar charts, with T-bars referring to standard deviations. *P-*values < 0.05 were regarded as statistically significant and were indicated by an asterisk (*). Cell cycle progression data from at least 10 000–30 000 events were analyzed using CXP software (Beckman Coulter South Africa (Pty) Ltd. (Pretoria, Gauteng, South Africa)). Cell cycle distributions was calculated with Cyflogic 1.2.1 released 2008/11/19 (Perttu Terho & Cyflo Ltd).

## Results

### Glucose and l-glutamine deprivation results in decreased cell density and round shrunken cells

**Fig. 1:**
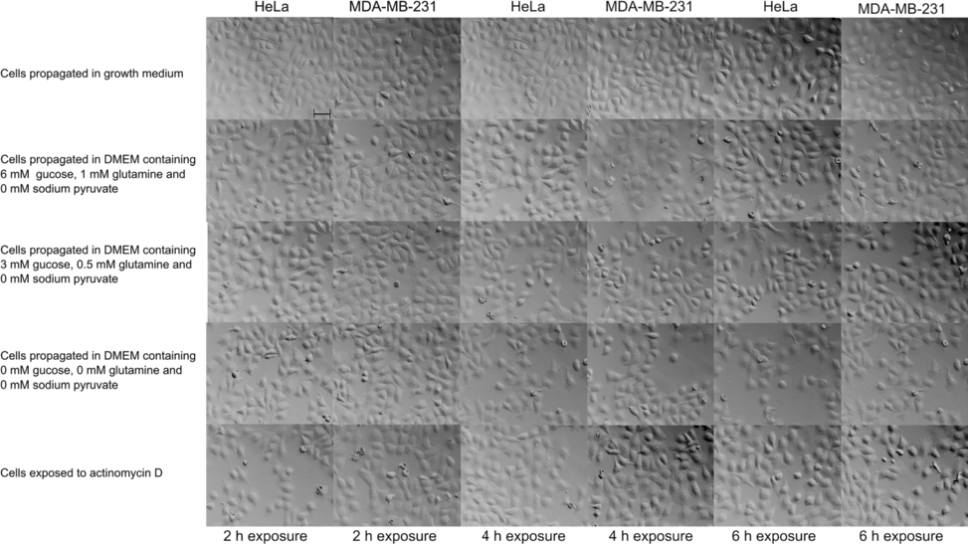
PlasDIC images after short-term exposures (2 h, 4 h, 6 h) glutamine- and glucose starvation. PlasDIC images of HeLa and MDA-MB-231 cells propagated in media according to metabolic state and cells exposed to actinomycin D for 2 h, 4 h and 6 h. Exposure to DMEM containing 6 mM glucose and 1 mM l-glutamine for 2 h resulted in decreased cell density. Exposure to DMEM containing 0 mM-3 mM glucose and 0 mM-0.5 mM l-glutamine for 2 h resulted in decreased cell density and rounded shrunken cells. After 4 h cells propagated in medium containing 6 mM glucose and 1 mM L-glutamine exhibited decreased cell density and rounded shrunken cells. Cells propagated in medium containing low quantities of glucose and glutamine or no glucose and glutamine for 4 h demonstrated decreasing cell density and increased number of cells appearing rounded and shrunken when compared to cells exposed to DMEM containing 6 mM glucose and 1 mM l-glutamine and cells propagated in growth medium. Cells propagated in DMEM containing 6 mM glucose and 1 mM l-glutamine for 6 h demonstrated decreased cell density and the presence of some rounded shrunken cells. The latter was also observed with cells propagated in DMEM containing 3 mM glucose and 0.5 mM l-glutamine for 6 h. The cells propagated in DMEM containing 0 mM glucose and 0 mM l-glutamine for 6 h also demonstrated the presence of some rounded shrunken cells and decreased cell density. The latter was more pronounced when compared to cells propagated in DMEM containing 3–6 mM glucose and 0.5-1 mM l-glutamine growth medium for 6 h. After 6 h exposure, cells were mostly present in interphase with cells still attached. The scale bar in all images represents 50 μm

Polarization-optical transmitted light differential interference contrast (PlasDIC) was used to demonstrate the effects of glucose-and glutamine deprivation on morphology in the cervical HeLa epithelial cell line and metastatic estrogen receptor negative breast cell line. Glucose-and glutamine deprivation, regardless of individual medium concentrations, for 2 h resulted in a slight decrease in cell density in both cell lines when compared to cells propagated in growth medium (Fig. 1). Cells were still attached with most cells present in metaphase. Rounded-and shrunken cells were also observed in cells propagated in medium with 0–3 mM glucose-and 0–0.05 mM glutamine. In addition, the lower the quantities of glucose-and glutamine, the lower the cell density when compared to cells propagated in growth medium, implying that glucose and glutamine both have essential roles in proliferation and morphology.

PlasDIC was also utilized to demonstrate the 4 h deprivation of glucose-and glutamine on cell morphology in HeLa-and MDA-MB-231 cells (Fig. 1). After 4 h exposure to DMEM containing 6 mM glucose and 1 mM l-glutamine, cell density is decreased with the presence of some rounded cells in both cell lines. Further reductions to 3 mM glucose and 0.5 mM l-glutamine accompanied with exposure for 4 h demonstrated decreased cell density and increased number of rounded and shrunken cells. Complete deprivation of glutamine and glucose for 4 h resulted in a further reduction in cell density accompanied with the presence of rounded and shrunken cells. However, all cells remained attached after exposure and the majority of cells occupied interphase. Furthermore, cell density was reduced more prominently after 4 h exposure to the metabolic media when compared to 2 h of exposure.

The effects of glutamine-and glucose deprivation on morphology were also investigated after 6 h (Fig. 1). Cells propagated in DMEM containing 3–6 mM glucose and 0.5-1 mM l-glutamine for 4 h period demonstrated decreased cell density and the presence of some rounded shrunken cells. The cells propagated in medium containing no glutamine and glucose also demonstrated the presence of some rounded shrunken cells and decreased cell density. The latter was more pronounced when compared to cells propagated in DMEM containing 3–6 mM glucose and 0.5-1 mM l-glutamine. As with the exposures for 2 h-and 4 h, cells were mostly present in interphase with cells still attached.

**Fig. 2:**
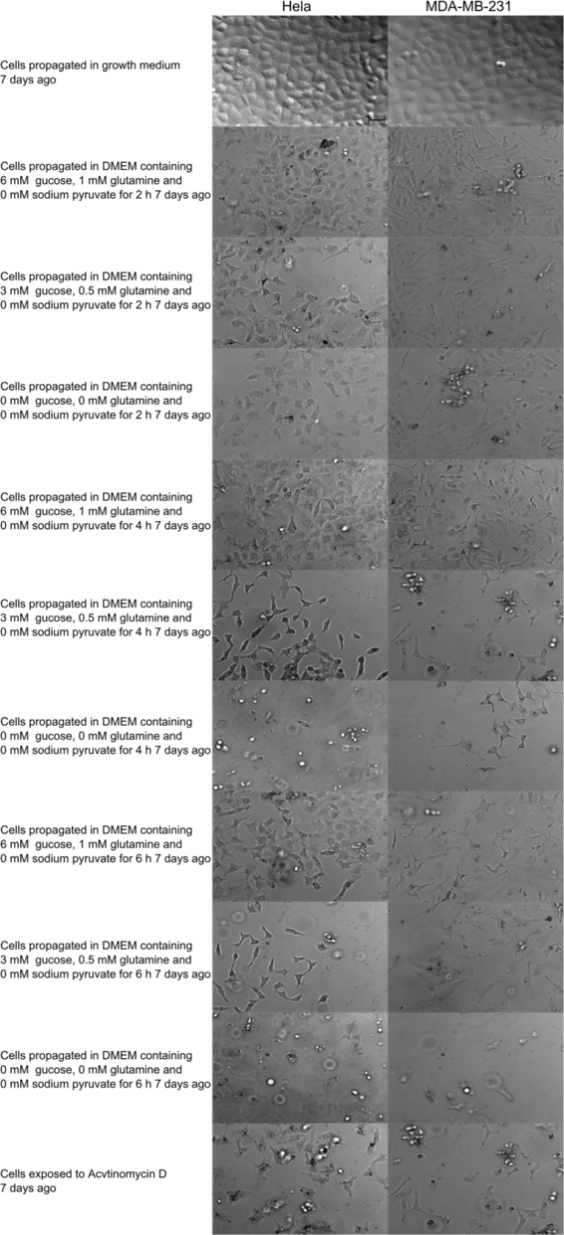
Unsuccessful rescue of HeLa and MDA-MB-231 after 7 days. HeLa and MDA-MB-231 cells propagated in media according to metabolic state for 2 h, 4 h and 6 h, 7 days ago, which after cells were washed and medium was replaced with DMEM containing 6 mM glucose and 1 mM l-glutamine. The effects of the decreasing l-glutamine and glucose concentrations were more pronounced 7 days after the exposure when compared to those on the day of exposure self. Effects on decreased cell density were also more severe. Morphological characteristics observed included decreased cell density, reduced cell size and elongation. All of the above-mentioned morphological characteristics were more prominent in the DMEM containing 0 mM glucose and 0 mM l-glutamine when compared to cells propagated in DMEM containing 3 mM-6 mM glucose and 0.5 mM-1 mM l-glutamine. The same applies for the 6 h exposures compared to the 2 h and 4 h exposures. However, all of the above-mentioned effects appeared earlier in the MDA-MB-213 cell line when compared to the HeLa cell line (20x magnification). The scale bar in all images represents 50 μm

Cell morphology was also investigated with regard to possible recuse and long term effects of typical physiological concentrations of glucose and L-glutamine. HeLa and MDA-MB-231 cells were exposed according to varying metabolic conditions for 2 h and 4 h and 6 h after which cells were washed and subsequently medium was replaced with DMEM containing 6 mM glucose and 1 mM l-glutamine for a 7 day period (Fig. 2). Morphology and cell density after 7 days presented with shrunken cells and elongated shape often accompanied with cell protrusions. MDA-MB-231-and HeLa cells propagated in medium containing 6 mM glucose and 1 mM l-glutamine for 2 h and 4 h, and allowed 7 days prior recovery demonstrated decreased cell size and the MDA-MB-231 cells were elongated. Cells propagated in DMEM containing 6 mM glucose and 1 mM l-glutamine for 6 h and allowed 7 days prior recovery demonstrated more prominent decreased cell size and elongated cell morphology in both cell lines. Cells exposed to medium containing 0–0.5 mM L-glutamine and 3 mM glucose also demonstrated decreased cell density and reduced cell size in both cell lines. The MDA-MB-231 cells exposed to these conditions also demonstrated elongated cells. This trend became more and more prominent with increased exposure to both metabolic conditions with both cell lines possessing elongated morphology at 4 h and 6 h of exposure after 7 days recovery when previously accompanied with unattached cells.

All the morphological characteristics of shrunken cell size and elongation increased with decreasing quantities of L-glutamine and glucose and longer exposure periods. However, all of the above-mentioned effects appeared earlier in the MDA-MB-213 cell line when compared to the HeLa cell line indicating that the metabolic states influences the highly metastatic cell line more prominently. Furthermore, this morphological data suggest that there are lasting effects after cells are deprived of nutrients, even after the medium was replaced with medium containing physiological concentrations of the nutrients.

### Dose-dependent glucose and l-glutamine deprivation results in apoptosis induction and cell cycle changes

**Fig. 3:**
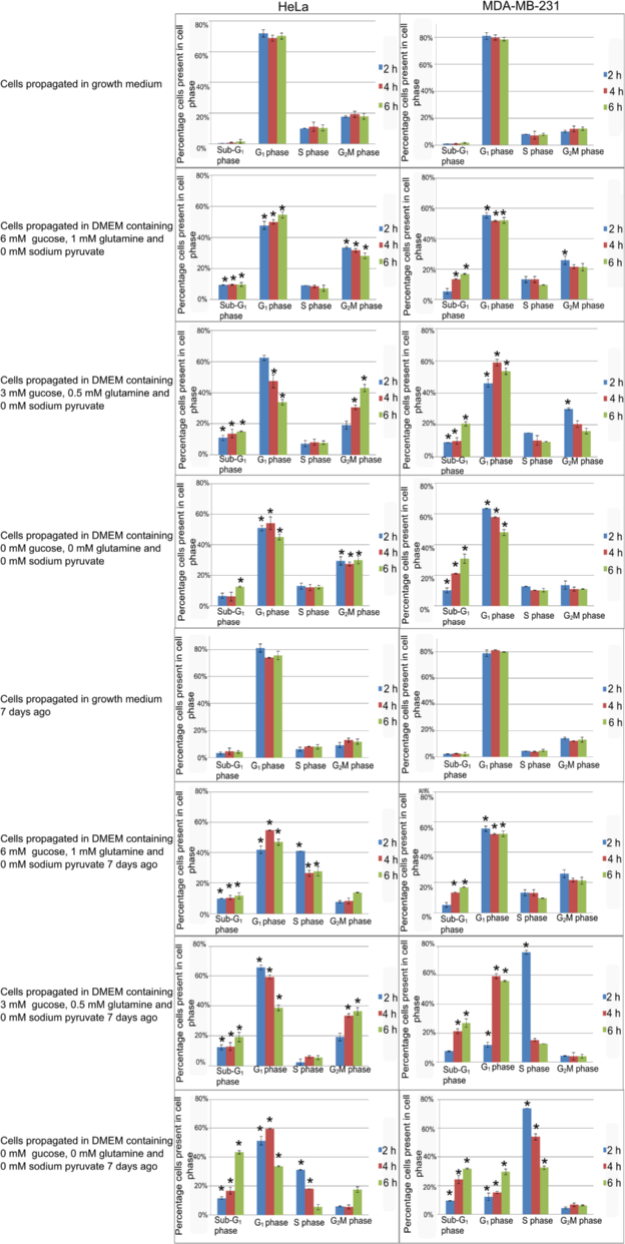
Cell cycle progression after partial- and complete glutamine- and glucose starvation for short term exposures and after 7 days. Percentage of cells occupying each cell cycle phase after cells was propagated in media according to metabolic state for the appropriate exposure period (2 h, 4 h and 6 h). Hela cells propagated in medium containing 6 mM glucose and 1 mM L-glutamine demonstrated an increased sub-G_1_- and G_2_M faction. MDA-MB-231 cells propagated in medium containing 6 mM glucose and 1 mM L-glutamine also showed an increased sub-G_1_ fraction. MDA-MB-231 and HeLa cells propagated in medium containing 6 mM glucose and 1 mM L-glutamine demonstrated an increased sub-G_1_ and G_2_M fraction. HeLa- and MDA-MB-231 cells propagated in media containing no glucose or L-glutamine demonstrated an increase in the number of apoptotic cells and the HeLa cells also showed an increase in the G_2_M fraction. The effects on the cell cycle and apoptosis induction on day 7 by means of flow cytometry using propidium iodide staining showed significant induction of apoptosis in all treated samples. Hela- and MDA-MB-231 cells propagated in medium containing 6 mM glucose and 1 mM L-glutamine 7 days before also demonstrated an increase in the number of cells occupying the S phase. The MDA-MB-231 cells also showed a G_2_M increase. The HeLa cells propagated in medium containing 3 mM glucose and 0.5 mM L-glutamine also presented with increased G_2_M fraction. MDA-MB-231 cells propagated in DMEM containing 0 mM-3 mM glucose and 0 mM-0.5 mM l-glutamine demonstrated an increased number of cells in the S phase. The Hela cells propagated in medium containing 0 mM glucose and 0 mM L-glutamine for 2 h and 4 h also showed an increased S phase. An asterisk (*) indicates *P*-value < 0.05

The influence of these different mediums on cell cycle progression was investigated using ethanol fixation, propidium iodide staining and flow cytometry. Exposure in Hela-and MDA-MB-231 cells to medium containing 6 mM glucose and 1 mM l-glutamine resulted in an increased number of cells in the sub-G_1_ phase, a decreased number of cells occupying the G_1_ phase and an increase in cells in the G_2_M phase with statistically insignificant changes between exposure for 2 h, 4 h and 6 h (Table 1 and Fig. 3). The changes in cell cycle progression were progressively more pronounced when cells were exposed to medium containing lower glucose-and l-glutamine quantities. In addition, the influence of the media according to metabolic state on cell cycle progression was progressively greater between different exposure periods when the media contains decreasing quantities of glucose and l-glutamine.

**Table 1:** Cell cycle progression histograms of HeLa and MDA-MB-231 cells propagated in media according to metabolic state for the appropriate exposure period (2 h, 4 h and 6 h) (*P*-value < 0.05)

Sample	Histogram profile
HeLa cells propagated in growth medium for 2 h	
HeLa cells propagated in growth medium for 4 h	
HeLa cells propagated in growth medium for 6 h	
HeLa cells propagated in medium containing 6 mM glucose and 1 mM L-glutamine for 2 h	
HeLa cells propagated in medium containing 6 mM glucose and 1 mM L-glutamine for 4 h	
HeLa cells propagated in medium containing 6 mM glucose and 1 mM L-glutamine for 6 h	
HeLa cells propagated in medium containing 3 mM glucose and 0.5 mM L-glutamine for 2 h	
HeLa cells propagated in medium containing 3 mM glucose and 0.5 mM L-glutamine for 4 h	
HeLa cells propagated in medium containing 3 mM glucose and 0.5 mM L-glutamine for 6 h	
HeLa cells propagated in medium containing 0 mM glucose and 0 mM L-glutamine for 2 h	
HeLa cells propagated in medium containing 0 mM glucose and 0 mM L-glutamine for 4 h	
HeLa cells propagated in medium containing 0 mM glucose and 0 mM L-glutamine for 6 h	
MDA-MB-231 cells propagated in growth medium for 2 h	
MDA-MB-231 cells propagated in growth medium for 4 h	
MDA-MB-231 cells propagated in growth medium for 6 h	
MDA-MB-231 cells propagated in medium containing 6 mM glucose and 1 mM L-glutamine for 2 h	
MDA-MB-231 cells propagated in medium containing 6 mM glucose and 1 mM L-glutamine for 4 h	
MDA-MB-231 cells propagated in medium containing 6 mM glucose and 1 mM L-glutamine for 6 h	
MDA-MB-231 cells propagated in medium containing 3 mM glucose and 0.5 mM L-glutamine for 2 h	
MDA-MB-231 cells propagated in medium containing 3 mM glucose and 0.5 mM L-glutamine for 4 h	
MDA-MB-231 cells propagated in medium containing 3 mM glucose and 0.5 mM L-glutamine for 6 h	
MDA-MB-231 cells propagated in medium containing 0 mM glucose and 0 mM L-glutamine for 2 h	
MDA-MB-231 cells propagated in medium containing 0 mM glucose and 0 mM L-glutamine for 4 h	
MDA-MB-231 cells propagated in medium containing 0 mM glucose and 0 mM L-glutamine for 6 h

Exposure to medium containing 3 mM glucose and 0.5 mM l-glutamine in Hela cells for 2 h resulted in 11 % of cells being present in sub-G_1_ phase, 63 % in the G_1_ phase, 7 % in the S phase, and 19 % in the G_2_M phase. However, after 4 h, 14 % of cells were in the sub-G_1_ phase, 48 % in the G_1_ phase, 8 % in the S phase, and 31 % in the G_2_M phase. After 6 h of exposure 15 % of cells were in the sub-G_1_ phase, 34 % in the G_1_ phase, 8 % in the S phase, and 43 % in the G_2_M phase. Exposure medium containing 3 mM glucose and 0.5 mM l-glutamine in MDA-MB-231 for 2 h resulted in 9 % of cells being present in the sub-G_1_ phase, 46 % in the G_1_ phase, 15 % in the S phase, and 30 % in the G_2_M phase. After exposure to medium containing 3 mM glucose and 0.5 mM l-glutamine for 4 h, 10 % of cells were in the Sub-G_1_ phase, 59 % in the G_1_ phase, 10 % in the S phase, and 21 % in the G_2_M phase. After 6 h of exposure 21 % of cells were in the sub-G_1_ phase, 54 % in the G_1_ phase, 10 % in the S phase, and 16 % in the G_2_M phase. These trends continued with the exposure of cells to medium containing 0 mM glucose and 0 mM l-glutamine.

In addition, the rescue and long-term effects of physiological concentrations of glucose and L-glutamine was also investigated. This was accomplished by exposing the cell lines to medium according to varying metabolic conditions for the appropriate period before cells were washed with PBS and medium was replaced with 6 mM glucose and 1 mM l-glutamine (medium was replaced every 2 days). The effects on the cell cycle and apoptosis induction on day 7 are demonstrated in Table 2 and Fig. 3. Cells pertaining to this exposure group demonstrated cell cycle progression data indicating significant apoptosis induction. The sub-G_1_ fractions increased with the decreasing glucose and l-glutamine quantities and increasing exposure periods (2 h, 4 h and 6 h).

**Table 2:** Cell cycle progression histograms of HeLa and MDA-MB-231 cells propagated in media according to metabolic state for the appropriate exposure period (2 h, 4 h and 6 h) 7 days ago, which after cells were washed and medium was replaced with condition 1 media (*P*-value < 0.05)

Sample	Histogram profile
HeLa cells propagated in growth medium for 2 h	
HeLa cells propagated in growth medium for 4 h	
HeLa cells propagated in growth medium for 6 h	
HeLa cells propagated in medium containing 6 mM glucose and 1 mM L-glutamine for 2 h	
HeLa cells propagated in medium containing 6 mM glucose and 1 mM L-glutamine for 4 h	
HeLa cells propagated in medium containing 6 mM glucose and 1 mM L-glutamine for 6 h	
HeLa cells propagated in medium containing 3 mM glucose and 0.5 mM L-glutamine for 2 h	
HeLa cells propagated in medium containing 3 mM glucose and 0.5 mM L-glutamine for 4 h	
HeLa cells propagated in medium containing 3 mM glucose and 0.5 mM L-glutamine for 6 h	
HeLa cells propagated in medium containing 0 mM glucose and 0 mM L-glutamine for 2 h	
HeLa cells propagated in medium containing 0 mM glucose and 0 mM L-glutamine for 4 h	
HeLa cells propagated in medium containing 0 mM glucose and 0 mM L-glutamine for 6 h	
MDA-MB-231 cells propagated in growth medium for 2 h	
MDA-MB-231 cells propagated in growth medium for 4 h	
MDA-MB-231 cells propagated in growth medium for 6 h	
MDA-MB-231 cells propagated in medium containing 6 mM glucose and 1 mM L-glutamine for 2 h	
MDA-MB-231 cells propagated in medium containing 6 mM glucose and 1 mM L-glutamine for 4 h	
MDA-MB-231 cells propagated in medium containing 6 mM glucose and 1 mM L-glutamine for 6 h	
MDA-MB-231 cells propagated in medium containing 3 mM glucose and 0.5 mM L-glutamine for 2 h	
MDA-MB-231 cells propagated in medium containing 3 mM glucose and 0.5 mM L-glutamine for 4 h	
MDA-MB-231 cells propagated in medium containing 3 mM glucose and 0.5 mM L-glutamine for 6 h	
MDA-MB-231 cells propagated in medium containing 0 mM glucose and 0 mM L-glutamine for 2 h	
MDA-MB-231 cells propagated in medium containing 0 mM glucose and 0 mM L-glutamine for 4 h	
MDA-MB-231 cells propagated in medium containing 0 mM glucose and 0 mM L-glutamine for 6 h	

Hela and MDA-MB-231 cells propagated in DMEM containing 6 mM glucose and 1 mM l-glutamine for 7 days demonstrated both an increase in apoptotic cells (sub-G_1_ phase) and an increase in the S phase. MDA-MB-231 cells also presented with an increased G_2_M phase when exposed to DMEM containing 6 mM glucose and 1 mM l-glutamine throughout. Hela cells propagated in DMEM containing 3 mM glucose and 0.05 mM l-glutamine and allowed 7 days recovery propagated in DMEM containing 6 mM glucose and 1 mM l-glutamine also presented with an apoptotic sub-G1 peak and an increased G_2_M phase. The MDA-MB-231 cells exposed to DMEM containing 3 mM glucose and 0.5 mM l-glutamine showed a very prominent increase in the S phase. All of the above-mentioned increases in number of cells occupying the respective cell cycle phases were associated with a corresponding decrease of cells occupying the G_1_ phase. HeLa cells exposed to DMEM containing no glucose or l-glutamine for short exposure periods and allowed 7 days recovery in DMEM containing 6 mM glucose and 1 mM l-glutamine showed an increase in the number of cells occupying the S phase. MDA-MB-231 cells exposed to DMEM containing no glucose or l-glutamine for short exposure periods and allowed 7 days recovery in DMEM containing 6 mM glucose and 1 mM l-glutamine resulted in an increase in the number of cells in the S phase; most prominently in the 2 h, followed by 4 h and lastly by 6 h accompanied by a steady increase in the number of apoptotic (sub-G_1_) cells and the number of cells in the G_1_ phase.

### Glucose and l-glutamine deprivation results in depolarisation of the mitochondrial membrane potential

**Fig. 4:**
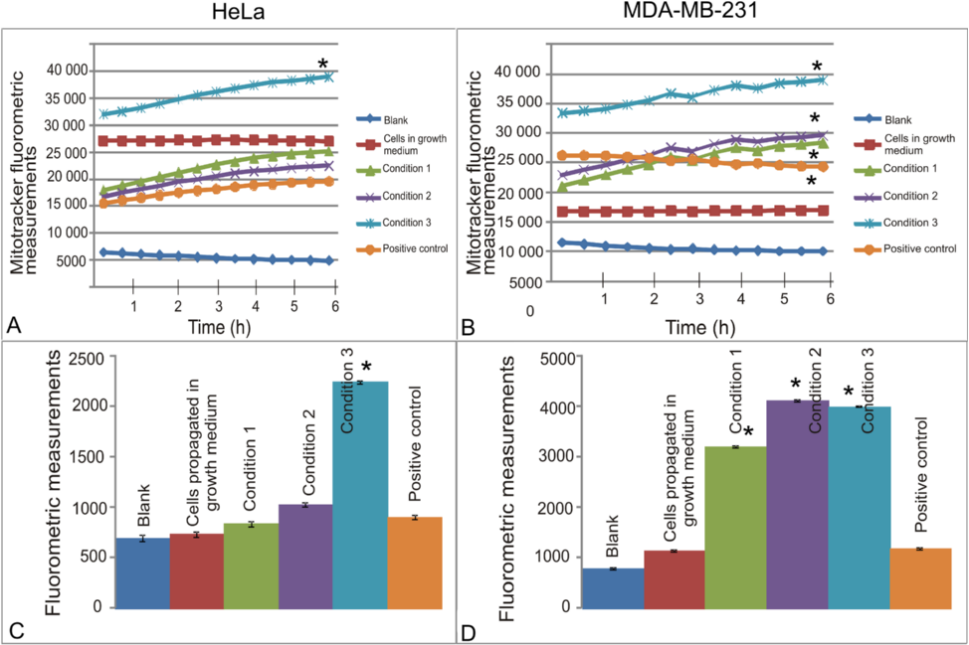
Mitochondrial membrane potential after partial- and complete glutamine- and glucose starvation for short term exposures and after 7 days. Mitochondrial membrane potential of HeLa cells (**a**) and MDA-MB-231 cells (**b**) exposed to medium consisting of varying metabolic conditions. Cells exposed to medium containing no glucose or l-glutamine were the only samples with reduced mitochondrial membrane potential indicating apoptosis induction. Fluorometrics and mitocapture demonstrated unsuccessful recovery and colony formation demonstrated that the HeLa (**c**) cell line was less prominently affected than the MDA-MB-231 (**d**) cell line. Little changes were observed in HeLa cells exposed to DMEM containing 3 mM-6 mM glucose and 0.5 mM-1 mM l-glutamine. However, cells exposed to medium containing 0 mM glucose and 0 mM L-glutamine demonstrated a statistically significant change in the mitochondrial membrane potential. With regard to the MDA-MB-231 cell lines, all three metabolic conditions affected the mitochondrial membrane potential cells exposed to DMEM containing 0 mM- 3 mM glucose and 0 mM-0.5 mM l-glutamine the most prominent. An asterisk (*) indicates *P*-value < 0.05

This study further investigated the induction of apoptosis by demonstrating the effect of the varying metabolic conditions on the mitochondrial membrane potential. The intrinsic apoptotic pathway involves the loss of mitochondrial membrane potential resulting in cytochrome *c* release and subsequent caspase activation [9]. The results indicated that the mitochondrial membrane potential of cells exposed to DMEM containing no glucose or l-glutamine was affected the most in both cells lines (Fig. 4a and b). In addition, MDA-MB-231 cells were also more prominently affected overall when compared to HeLa cells. Recovery and colony formation after 7 days propagation in DMEM containing 6 mM glucose and 1 mM l-glutamine indicated that MDA-MB-231 cells are still more prominently affected (Fig. 4c and d). HeLa cells were also presenting with a significant number of cells possessing a reduced mitochondrial membrane potential after exposure to medium containing no glucose or l-glutamine, even 7 days after withdrawing the media and replacing with DMEM containing 6 mM glucose and 1 mM l-glutamine. MDA-MB-231 was affected with any variance in the metabolic state of the media with DMEM containing 0–3 mM glucose and 0–0.5 mM l-glutamine resulting in the most prominently presented reduced mitochondrial membrane potential.

### Unsuccessful cell recovery demonstrating increased hydrogen peroxide generation

**Fig. 5:**
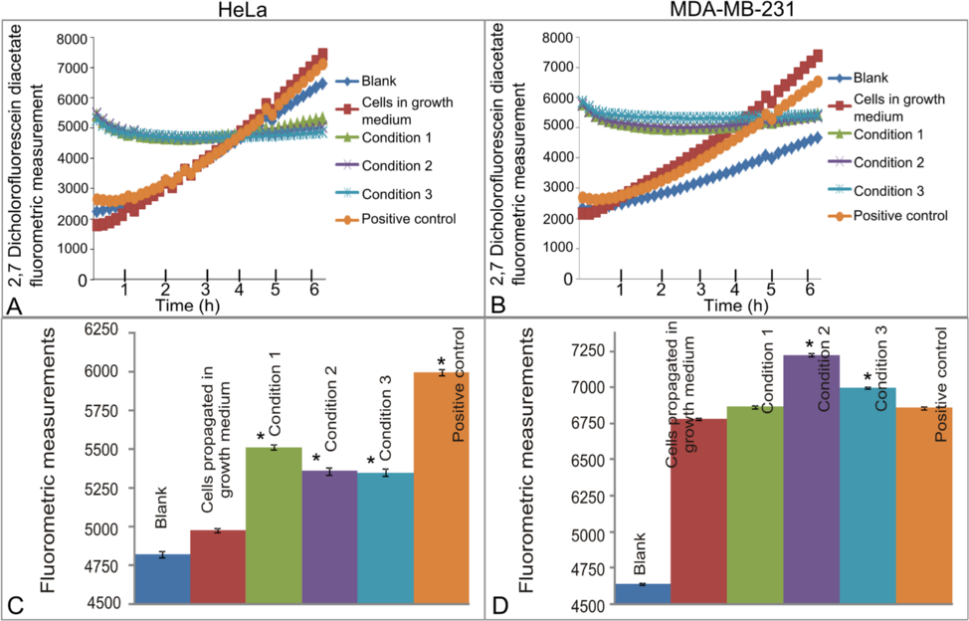
Hydrogen peroxide production after partial- and complete glutamine- and glucose starvation for short term exposures and after 7 days. Hydrogen peroxide generation in HeLa cells (**a**) and MDA-MB-231 (**b**) after exposure to media presenting with different metabolic conditions did not change in any statistically significant manner (*P*-value > 0.05). In addition, cell recovery by propagating exposed HeLa cells (**c**) and MDA-MB-231 (**d**) in DMEM containing 6 mM glucose and 1 mM l-glutamine for 7 days was unsuccessful and demonstrating increased hydrogen peroxide production. An asterisk (*) indicates *P*-value < 0.05

Hydrogen peroxide production was determined by means of a DCFDA which, upon oxidation by ROS and peroxides, is converted to the highly fluorescent derivative DCF [21, 22]. Exposure to the various mediums did not change the hydrogen peroxide production in the first six hours after exposure (Fig. 5a and b). However, the results from the cell lines allowed 7 days recovery after 7 days propagation in DMEM containing 6 mM glucose and 1 mM l-glutamine demonstrated that the effects of the different metabolic media still affected the cell functioning and ROS production after being replaced with DMEM containing 6 mM glucose and 1 mM l-glutamine as indicated by the increased ROS production in all cell lines exposed to the varied metabolic mediums (Fig. 5c and d). In the HeLa cell line, ROS production was increased more prominently when exposed to DMEM containing 6 mM glucose and 1 mM l-glutamine followed by decreasing glucose- and glutamine amounts. Results also indicated that MDA-MB-231 cells propagated in growth medium produced larger quantities of ROS possibly owing to their highly glycolytic- and metastatic nature. However, the metabolic media also increased their ROS production, most prominently by DMEM containing 3 mM glucose and 0.05 mM l-glutamine.

### Unsuccessful cell recovery demonstrating increased lysosomal staining

**Fig. 6:**
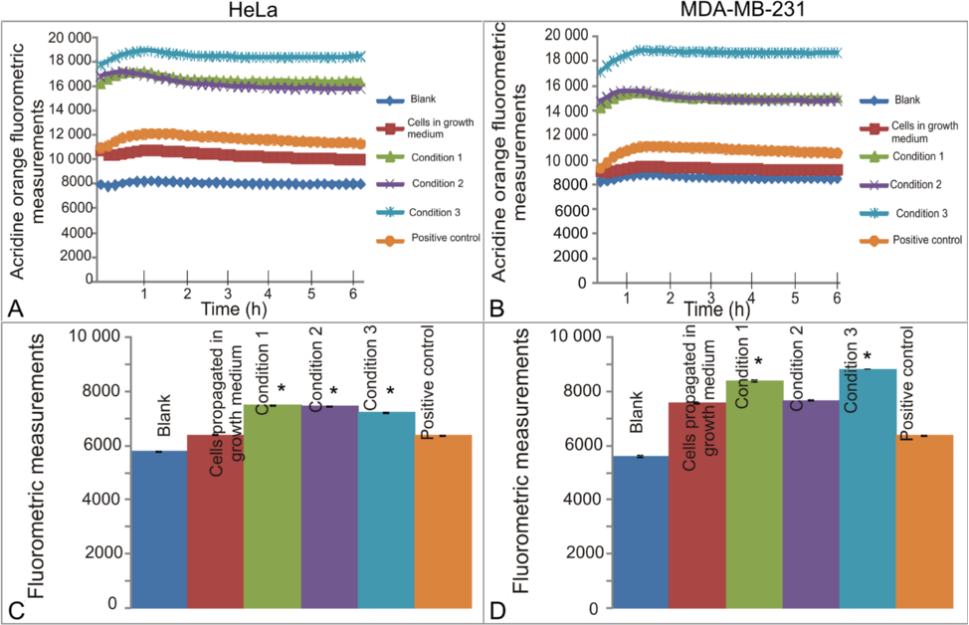
Acridine orange staining after partial- and complete glutamine- and glucose starvation for short term exposures and after 7 days. Acridine orange staining of HeLa cells (**a**) and MDA-MB-231 (**b**) after exposure to media presenting with different metabolic conditions. Acridine orange production did not change in any statistically significant manner when exposed to these metabolic conditions (*P*-value > 0.05). Acridine orange staining and colony formation after 7 days in HeLa (**c**) and MDA-MB-231 cells (**d**). Results demonstrated that glucose-and glutamine deprivation effects cell functioning days after exposure was terminated with all the exposure to conditioned media demonstrating increased acridine orange staining suggesting an increase in acidity. An asterisk (*) indicates *P*-value < 0.05

Acridine orange is a lysosomotropic fluorescent compound that moves freely across cell membranes when uncharged. However, acridine orange accumulates in its protonated forming acidic compartments and thus serves as a tracer for acidic vesicular organelles including autophagic vacuoles and lysosomes [23]. The initial 6 h exposure to the medium consisting of various metabolic states did not result in a significant increased lysosomal staining in either cell line (Fig. 6a and b). Cells lines that were exposed to various metabolic states followed by 7 days recovery in DMEM containing 6 mM glucose and 1 mM l-glutamine demonstrated increased lysosomal staining indicating an increase in acidic compartments and vacuole formation (Fig. 6c and d).

## Discussion

Cancer cells exert several intrinsic gene mutations (affecting p53, MYC, AMPK, PI3K and HIF1) and extrinsic responses to the tumour environment (hypoxia and acidity). This results in an altered cellular metabolism generating high amounts of ATP resulting in enhanced proliferation, increased macromolecule synthesis and the balance of a delicate redox homeostasis [2].

Many tumorigenic cells metabolize much higher amounts of glucose than lactate in aerobic glycolysis. This is known as the Warburg effect and this occurs even in the presence of mitochondrial respiration [24]. A major source of energy in cell metabolism is glucose that is metabolised to pyruvate in glycolysis and further oxidized to form carbon dioxide generating ATP in the tricarboxylic acid cycle. Glutamine metabolism contributes to biomass precursors as it is a major carbon and nitrogen source [25].

This study investigated the necessity of glucose and l-glutamine in cell proliferation and functioning. This was accomplished by demonstrating the effects of media with different metabolic states, containing decreasing glucose or l-glutamine concentrations, in a human cervical adenocarcinoma cell line (HeLa) and a highly metastatic breast adenocarcinoma cell line (MDA-MB-231) on morphology, cell cycle progression, hydrogen peroxide generation and cell death via apoptosis- and autophagy induction.

Morphological findings demonstrate that decreasing amounts of glucose and l-glutamine in the growth medium resulted in decreased cell density in both cell lines. These effects were more pronounced 7 days after the exposure medium was replaced with medium containing 6 mM glucose and 1 mM L-glutamine. Since glucose is an essential source of carbon and energy required for cell survival and proliferation, glucose deprivation results in a failure of glycolytic enzyme induction with subsequent cessation of cell division [26]. Glucose deprivation was also found to induce cytotoxicity in the multidrug-resistant carcinoma cell line (MCF-7/ADR). Within 10 min, several signaling pathways including extracellular regulated protein kinases (ERK1/2), Lyn Kinase (a *src* family kinase) and c-Jun N-terminal kinase (JNK) were activated. ERK1/2 activation results in subsequent increased ROS generation. ERK1/2 are members of the mitogen activated protein kinase (MAPK) family [27]. MAPK is responsible for the phosphorylation of nuclear substrates including redox regulated transcription factors (*c*-Myc and *c*-fos) involved in cellular responses to oxidative stress [28].

Cell progression data suggested that glucose- and glutamine deprivation resulted in a G_2_M block after 2–6 h in the HeLa cell line and 2–4 h in the MDA-MB-231 cell line accompanied with an increased induction of apoptosis in both cell lines. Glucose deprivation in a non-leukemic murine cell line (32Dcl23), transformed 32Dcl23 cell line and fibrosarcoma cell line (KHT-C2-LP1) also resulted in G_2_M arrest [29, 30]. Further, it has been widely reported that glutamine deprivation results in G_1_ cell cycle arrest in non-transformed cells. Glutamine deprivation in a human hepatocarcinoma (Hep3B) cell line resulted in significant altered gene expression related to the G_2_M phase and DNA damage checkpoint including growth arrest and DNA-damage-inducible 45, gamma (GADD45G) [31]. Abcouver, et al. [32] also reported that glutamine deprivation in primary ductal carcinoma breast cells (TSE) and breast epithelial cells (HBL) resulted in rapid increase in GADD45 and DNA damage-inducible transcript 3 (GADD153). Cell cycle progression data also demonstrated an increased number of cells occupying the G_2_M phase after glutamine deprivation. K-Ras-driven tumorigenic cells arrest in either the S phase or G_2_M phase upon glutamine deprivation [32, 33]. Glutamine or glucose deprivation also results in synthetic lethality of various antitumor cell phase specific compounds. For example, glutamine deprivation in combination with capecitabine or paclitaxel resulted in enhanced cell death [5]. The increase of cells occupying G_1_ phase after exposure to growth media containing decreased glutamine and glucose quantities suggests apoptosis induction. Various reports verify the induction of apoptosis by glucose or glutamine deprivation observed here including human embryonic kidney cells (HA1E) and mouse embryonic fibroblast cell line (NIH3T3) [34].

During the intrinsic apoptotic pathway, cellular stress signaling leads to permeabilization of the mitochondrial outer membrane resulting cytochrome *c* release into the cytosol caspase activation [31]. This is supported by previous reports where glucose deprivation in hemapoietic FL5.12 cells, human- and rat neutrophils resulted in mitochondrial membrane potential depolarisation [35, 36]. Thus, apoptosis induction was verified by additional flow cytometry findings demonstrating that exposure to the medium containing decreased glucose and l-glutamine quantities resulted in a reduction of the mitochondrial membrane potential. Mitochondrial ultrastructure studies revealed that complete glucose starvation for 6 h-9 h resulted in increased mitochondrial fragmentation, while reduced glucose or complete glutamine starvation resulted in mitochondrial elongation. Simultaneously, starvation of glucose and glutamine also lead to fused mitochondria [35]. Glutamine deprivation in human lung carcinoma cells (A549) demonstrated dense mitochondria with no further abnormal mitochondrial structural changes. Glutamine supplementation (1 mM) resulted in an increase of mitochondria number accompanied with large mitochondria size. The potential sensitive chloromethyltetramethylrosamine (CMTMRos) stain in the same study demonstrated that complete glutamine deprivation resulted in less round mitochondria and not as densely located around the nucleus when compared to cells cultured in medium containing 1 mM glutamine. Mitochondria in both conditions possessed thin filamentous structures [36].

Cytochrome *c* release is another hallmark of the intrinsic apoptotic pathway and was observed after glutamine deprivation in FL5.12 cells, KB26.5 murine hybridomas [37, 38]. Another study reported that glutamine deprivation in murine hybridoma cell line (Sp20) lead to the release of cytochrome *c* and SMAC/DIABLO accompanied with Bax translocation to the mitochondria and caspase 9 activation which is all characteristics of the intrinsic pathway [39]. Further, glutamine deprivation in myc-transformed fibroblasts has demonstrated that bcl-2 and caspase 9 inhibition prevented cell death verifying the induction of the intrinsic pathway [37]. Glutamine deprivation in the lymphoblastic leukemia CD4^+^ (CEM clone 13) cells, human lymphoblastic leukeumia (CEM-CCRF) cells and human promyeloblast (HL-60) cells also led to cell shrinkage and activation of CD95 receptors suggesting extrinsic pathway involvement [40].

This study demonstrated that glucose- and l-glutamine deprivation resulted in increased hydrogen peroxide production after a 7 day recovery period in both cell lines. An environment low in glucose results in increased oxidative phosphorylation for adequate ATP supply; this would lead to an additional oxygen electron reduction (electron transport chain) with subsequent increased ROS. The increased ROS (hydrogen peroxide and superoxide) is due to less reduced nicotinamide adenine dinucleotide- and pyruvate production in the pentose phosphate system and glycolysis, respectively [41].

Several reports have also indicated that glucose deprivation also caused increased hydrogen peroxide production in several cell lines including Chinese hamster ovary cell line (CHO), human liver carcinoma cell line (Hep2G), human pancreatic carcinoma (PANC1) and human fibroblasts [42–45]. Glucose deprivation resulting in oxidative stress also increased association between death associated protein 6 (DAXX), apoptosis signal-regulating kinase 1 (ASK1) and DAXX relocation from the nucleus to the cytoplasm. This is of importance since DAXX mediates the recruitment of ASK1 to FAS necessary for Fas-mediated apoptosis. [44]. Owada, et al. [43] also demonstrated that increased hydrogen peroxide resulting from glucose deprivation in Hep2G and PANC1 cells demonstrated subsequent AKT phosphorylation and activation [43, 45].

## Conclusion

This study demonstrated that exposure to decreasing quantities glucose or l-glutamine resulted in decreased cell density, G_2_M block and apoptosis induction within a few hours. After 7 days of being cultured in DMEM containing 6 mM glucose and 1 mM l-glutamine the above-mentioned effects are more prominent accompanied with a reduction in the mitochondrial membrane potential and increased acridine orange staining in both cell lines. In addition, the highly glycolytic- and metastatic cell line was more prominently affected when compared to the cervical carcinoma cell line. This study thus contributes the knowledge regarding the *in vitro* effects and signal transduction of glucose or l-glutamine deprivation in tumorigenic cell lines. Further research is imperative since it can potentially identify new targets for chemotherapy in the ongoing battle against cancer.
